# *trans*-Bis[bis­(di­phenyl­phosphan­yl)methane-κ^2^ *P*,*P*′]di­chlorido­ruthenium(II): a triclinic polymorph

**DOI:** 10.1107/S2414314623009847

**Published:** 2023-11-14

**Authors:** Monsuru T. Kelani, Alfred Muller, Koop Lammertsma

**Affiliations:** a Department of Chemical Sciences, University of Johannesburg, 2006, South Africa; Vienna University of Technology, Austria

**Keywords:** crystal structure, ruthenium, dimorphism, bidentate ligand

## Abstract

The title compound, [RuCl_2_(C_25_H_22_P_2_)_2_] or [RuCl_2_(dppm)_2_] (dppm = bis­(di­phenyl­phosphan­yl)methane, C_25_H_22_P_2_) crystallizes as two half-mol­ecules (completed by inversion symmetry) in space group *P*  (*Z* = 2), with the Ru^II^ atoms occupying inversion centers at 0,0,0 and 1/2, 1/2, 1/2, respectively. The bidentate phosphane ligands occupy equatorial positions while the chlorido ligands complete the distorted octa­hedral coordination spheres at axial positions. The bite angles of the phosphane chelates are similar for the two mol­ecules [(P—Ru—P)_*avg.*_ = 71.1°], while there are significant differences in the twisting of the methyl­ene backbone, with a distance of the methyl­ene C atom from the RuP_4_ plane of 0.659 (2) and 0.299 (3) Å, respectively, and also for the phenyl substituents for both mol­ecules due to variations in weak C—H⋯Cl inter­actions.

## Abstract 2

The title compound crystallizes with two different inversion-symmetric mol­ecules having a distorted octa­hedral {P_4_Cl_2_} coordination environment, and an average P—Ru—P bite angle of 71.1°.

## Graphical abstract

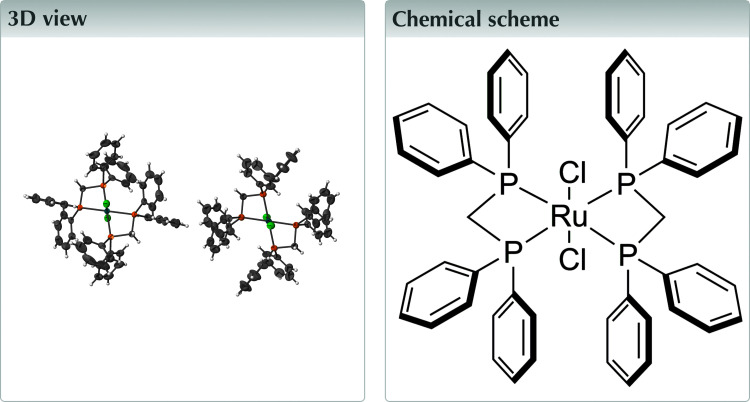

## Structure description

Ruthenium complexes have proven versatility in catalysis (Younus *et al.*, 2015 ▸; Saha *et al.*, 2022 ▸) and anti-cancer therapy (Levina *et al.*, 2009 ▸). Hence, the quest for contributions towards advancing the exploration of ruthenium-based complexes in coordination chemistry is still on-going. Moreover, in the context of advancing sustainability with inexpensive materials, ruthenium(II) phosphane complexes are widely used as catalysts, *e.g.* in the hydrogenation of carbon dioxide to methanol (Wesselbaum *et al.*, 2012 ▸), and formic acid (Tai *et al.*, 2002 ▸), as well as for the homogeneous catalytic degradation of the latter (Treigerman & Sasson, 2017 ▸).

The title compound was reported previously, crystallizing as a monoclinic ansolvate (Chakravarty *et al.*, 1984 ▸). Moreover, various solvated forms are also known: a triclinic *N*,*N*-di­methyl­formamide solvate (Treigerman & Sasson, 2017 ▸), a triclinic di­chloro­methane acetone solvate hemihydrate (Figueira *et al.*, 2006 ▸), and a triclinic dideuterodi­chloro­methane solvate (Lynam *et al.*, 2008 ▸). We report here the triclinic polymorph of the ansolvate.

**Figure 1:**
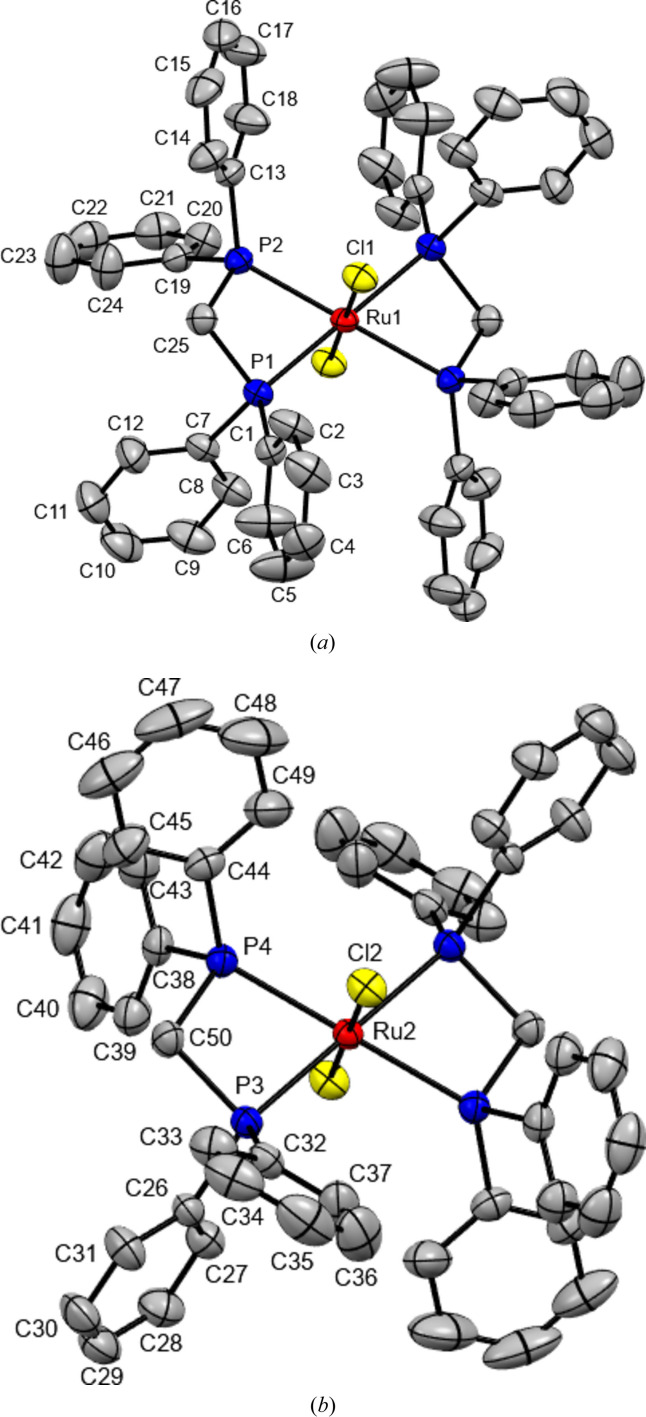
The mol­ecular structures of the two mol­ecules in the title compound. Displacement ellipsoids are drawn at the 50% probability level (H atoms were removed for clarity). Non-labelled atoms are generated by inversion symmetry (symmetry operations: −*x*, −*y*, −*z* for mol­ecule Ru1; −*x* + 1, −*y* + 1, −*z* + 1 for mol­ecule Ru2).

**Figure 2:**
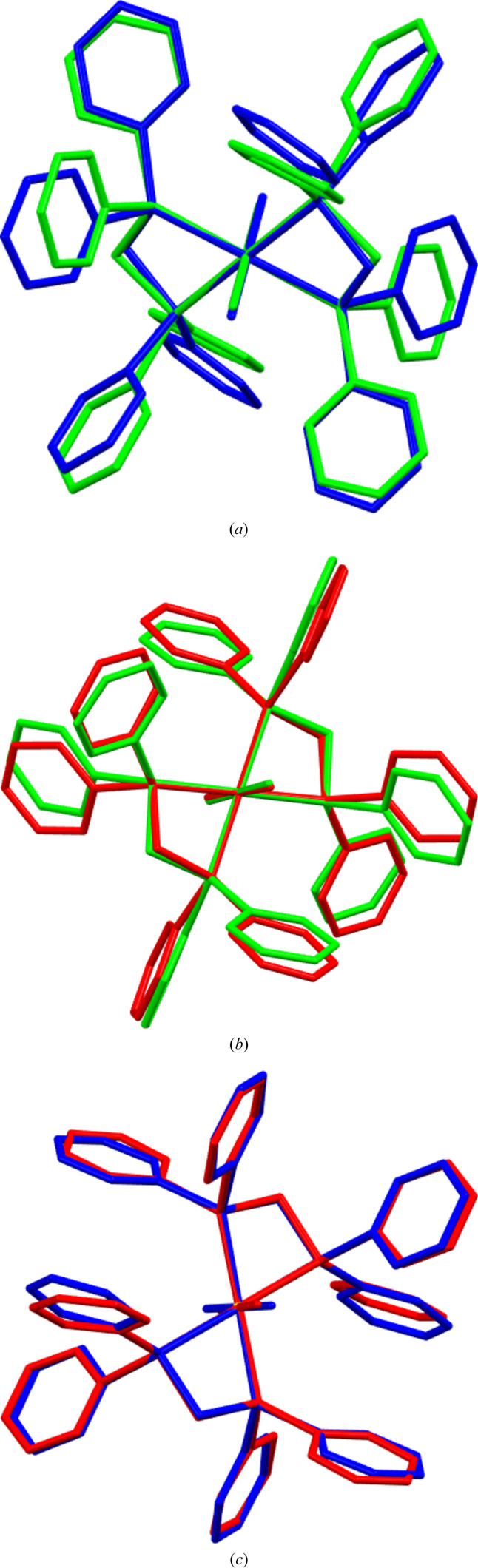
(*a*) Overlay of the two mol­ecules Ru1 (blue) and Ru2 (green) of the title compound; (*b*) overlay of mol­ecule Ru1 of the title compound (blue) and that of the monoclinic polymorph (red); (*c*) overlay of mol­ecule Ru2 of the title compound (green) and that of the monoclinic polymorph (red).

**Figure 3:**
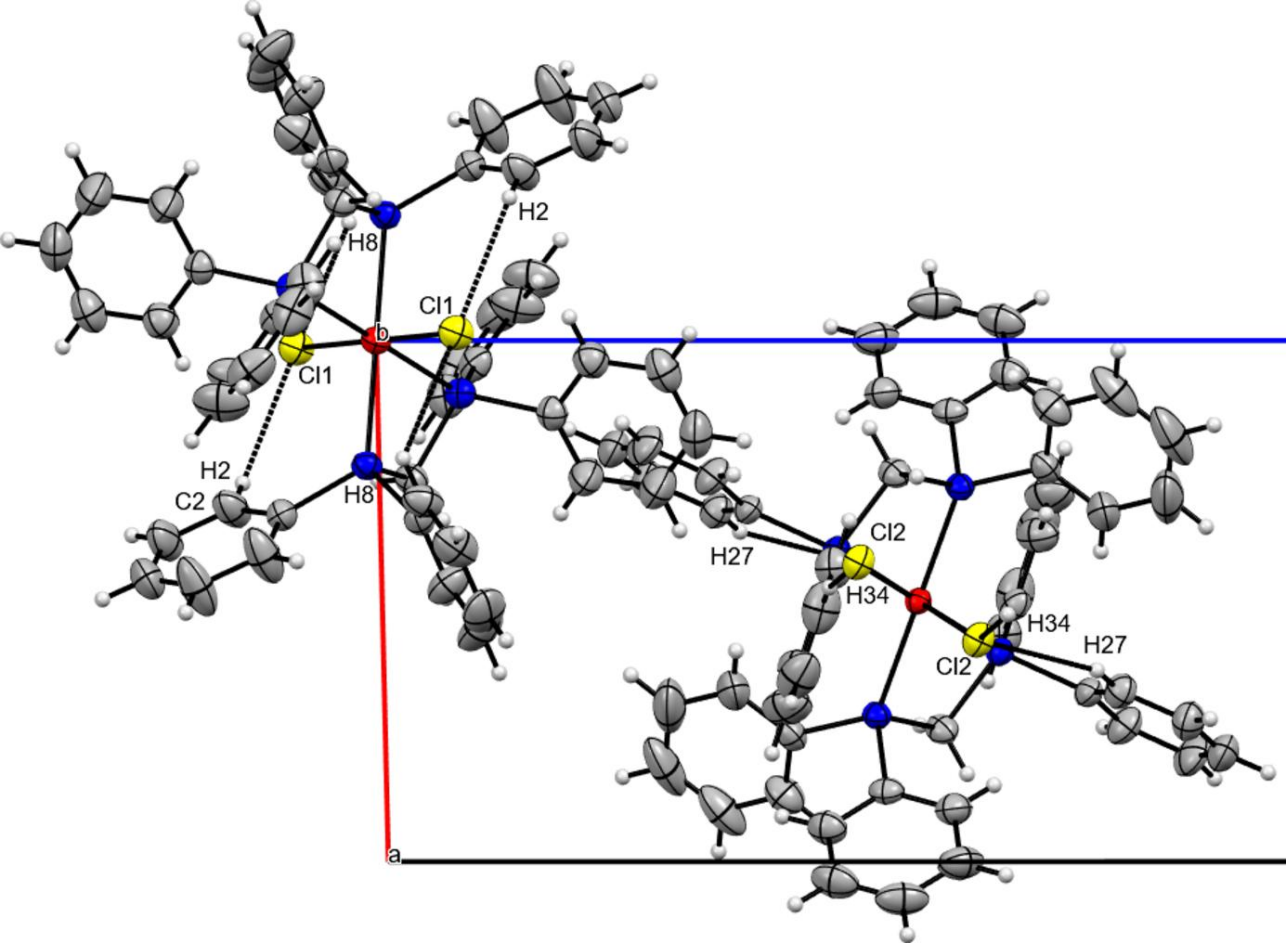
Packing plot in a view along [100] and selected hydrogen-bonding inter­actions (dashed lines) of the title compound.

The asymmetric unit of the title compound comprises two half-mol­ecules (Fig. 1 ▸), with the Ru^II^ atoms situated at inversion centers (at 0,0,0 and 1/2, 1/2, 1/2). Bond lengths and angles of the Ru^II^ coordination spheres (Table 1 ▸) are within the range of the monoclinic polymorph (Chakravarty *et al.*, 1984 ▸) or the solvated triclinic solvates (Treigerman & Sasson, 2017 ▸; Figueira *et al.*, 2006 ▸; Lynam *et al.*, 2008 ▸). Fig. 2 ▸ *a* shows the overlay of the two mol­ecules present in the title compound; the root-mean-square deviation (r.m.s.d.) between the two mol­ecules is 0.6828 Å. The non-solvated monoclinic polymorph and the title compound appear to be closely related as both have mol­ecules situated at inversion centers, albeit there are two independent special positions for the title compound *versus* the one of the reported monoclinic polymorph. Further to this, a comparative overlay of the mol­ecules in the two polymorphs, *i.e*. each of the two independent mol­ecules of the title compound overlayed with that of the reported monoclinic polymorph (Fig. 2 ▸ *b*,*c*), reveals differences in the orientations of some phenyl rings; r.m.s.d. are 0.3079 Å for overlays of mol­ecule Ru1 of the title compound and that of the monoclinic polymorph, and 0.4154 Å for overlays of mol­ecule Ru2 of the title compound and that of the monoclinic polymorph. The inversion symmetry of all mol­ecules in the triclinic title polymorph and the monoclinic polymorph causes a *trans* configuration of all ligands in the octa­hedral coordination environment, with the bis-phosphane ligands chelating in equatorial positions and the Cl ligands situated at axial positions. Most noticeable are the bite angles (P—Ru—P)_*avg.*_ of 71.1° in the title compound, causing a considerable distortion of the ideal octa­hedral environment. Inter­estingly, the methyl­ene backbone is twisted out from the equatorial plane differently for the two mol­ecules [distance of the C atom from the RuP_4_ plane 0.659 (2) Å, dihedral angle between the P—C—P plane and the equatorial plane 31.31 (10)° for mol­ecule Ru1 and 0.299 (3) Å and 14.00 (10)°, respectively, for mol­ecule Ru2]. This may be due to the different intra- and inter­molecular C—H⋯Cl inter­actions, which consolidate the crystal packing in the title compound (Table 2 ▸, Fig. 3 ▸).

## Synthesis and crystallization

Bis(di­phenyl­phosphan­yl)methane (30 mg, 0.08 mmol, 2 eq.) was added to a solution of the di­chlorido­(*η* ^6^-benzene)­ruthenium(II) dimer (20 mg, 0.04 mmol, 1 eq.) in methanol at room temperature for 24 h with continuous stirring. Yellow crystals of the title compound were obtained by slow evaporation of the solvent.

## Refinement

Crystal data, data collection, and structure refinement details are summarized in Table 3 ▸.

## Supplementary Material

Crystal structure: contains datablock(s) I. DOI: 10.1107/S2414314623009847/wm4201sup1.cif

Structure factors: contains datablock(s) I. DOI: 10.1107/S2414314623009847/wm4201Isup3.hkl

CCDC reference: 2307000

Additional supporting information:  crystallographic information; 3D view; checkCIF report

## Figures and Tables

**Table 1:** Selected geometric parameters (Å, °)

P1—Ru1	2.3623 (12)	P4—Ru2	2.3529 (11)
P2—Ru1	2.3573 (9)	Cl1—Ru1	2.4426 (10)
P3—Ru2	2.3882 (9)	Cl2—Ru2	2.4375 (11)
			
P2^i^—Ru1—P2	180.0	P4^ii^—Ru2—P3	108.09 (3)
P2^i^—Ru1—P1	109.78 (3)	P4—Ru2—P3	71.91 (3)
P2—Ru1—P1	70.22 (3)	P3^ii^—Ru2—P3	180.0
P2^i^—Ru1—Cl1	95.43 (4)	P4^ii^—Ru2—Cl2	85.27 (3)
P2—Ru1—Cl1	84.58 (4)	P4—Ru2—Cl2	94.73 (3)
P1—Ru1—Cl1	86.47 (3)	P3^ii^—Ru2—Cl2	82.43 (3)
P1^i^—Ru1—Cl1	93.53 (3)	P3—Ru2—Cl2	97.57 (3)

Symmetry codes: (i) 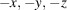 ; (ii) 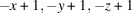 .

**Table 2:** Hydrogen-bond geometry (Å, °)

*D*—H⋯*A*	*D*—H	H⋯*A*	*D*⋯*A*	*D*—H⋯*A*
C8—H8⋯Cl1^i^	0.93	2.75	3.302 (3)	119
C34—H34⋯Cl2^iii^	0.93	2.91	3.813 (3)	165
C2—H2⋯Cl1	0.93	2.75	3.429 (3)	131
C27—H27⋯Cl2	0.93	2.66	3.506 (3)	152

Symmetry codes: (i) 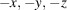 ; (iii) 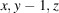 .

**Table 3:** Experimental details

Crystal data	
Chemical formula	[RuCl_2_(C_25_H_22_P_2_)_2_]
*M*_r_	940.70
Crystal system, space group	Triclinic, *P* 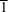
Temperature (K)	273
*a*, *b*, *c* (Å)	10.261 (5), 11.243 (5), 20.198 (9)
α, β, γ (°)	84.857 (15), 87.185 (16), 72.525 (15)
*V* (Å^3^)	2212.8 (17)
*Z*	2
Radiation type	Mo *K*α
μ (mm^−1^)	0.65
Crystal size (mm)	0.21 × 0.11 × 0.08

Data collection	
Diffractometer	Bruker APEXII CCD
Absorption correction	Multi-scan (*SADABS*; Krause *et al.*, 2015 ▸)
*T*_min_, *T*_max_	0.665, 0.746
No. of measured, independent and observed [*I* > 2σ(*I*)] reflections	99624, 10851, 8574
*R*_int_	0.072
(sin θ/λ)_max_ (Å^−1^)	0.667

Refinement	
*R*[*F* ^2^ > 2σ(*F* ^2^)], *wR*(*F* ^2^), *S*	0.034, 0.083, 1.04
No. of reflections	10851
No. of parameters	517
H-atom treatment	H-atom parameters constrained
Δρ_max_, Δρ_min_ (e Å^−3^)	0.49, −0.36

Computer programs: *APEX2* and *SAINT* (Bruker, 2010 ▸), *SHELXT* (Sheldrick, 2015*a* ▸), *SHELXL* (Sheldrick, 2015*b* ▸), *Mercury* (Macrae *et al.*, 2020 ▸), *WinGX* (Farrugia, 2012 ▸) and *publCIF* (Westrip, 2010 ▸).

## full crystallographic data

### Crystal data

**Table:**

[RuCl_2_(C_25_H_22_P_2_)_2_]	*Z* = 2
*M**_r_* = 940.70	*F*(000) = 964
Triclinic, *P*1	*D*_x_ = 1.412 Mg m^−^^3^
*a* = 10.261 (5) Å	Mo *K*α radiation, λ = 0.71073 Å
*b* = 11.243 (5) Å	Cell parameters from 9422 reflections
*c* = 20.198 (9) Å	θ = 2.3–27.6°
α = 84.857 (15)°	µ = 0.65 mm^−^^1^
β = 87.185 (16)°	*T* = 273 K
γ = 72.525 (15)°	Block, yellow
*V* = 2212.8 (17) Å^3^	0.21 × 0.11 × 0.08 mm

### Data collection

**Table:**

Bruker APEXII CCD diffractometer	8574 reflections with *I* > 2σ(*I*)
φ and ω scans	*R*_int_ = 0.072
Absorption correction: multi-scan (SADABS; Krause *et al.*, 2015)	θ_max_ = 28.3°, θ_min_ = 1.9°
*T*_min_ = 0.665, *T*_max_ = 0.746	*h* = −13→13
99624 measured reflections	*k* = −14→14
10851 independent reflections	*l* = −26→26

### Refinement

**Table:**

Refinement on *F*^2^	0 restraints
Least-squares matrix: full	Hydrogen site location: inferred from neighbouring sites
*R*[*F*^2^ > 2σ(*F*^2^)] = 0.034	H-atom parameters constrained
*wR*(*F*^2^) = 0.083	*w* = 1/[σ^2^(*F*_o_^2^) + (0.0316*P*)^2^ + 0.9131*P*] where *P* = (*F*_o_^2^ + 2*F*_c_^2^)/3
*S* = 1.04	(Δ/σ)_max_ < 0.001
10851 reflections	Δρ_max_ = 0.49 e Å^−^^3^
517 parameters	Δρ_min_ = −0.36 e Å^−^^3^

### Special details

**Table:**

Geometry. All esds (except the esd in the dihedral angle between two l.s. planes) are estimated using the full covariance matrix. The cell esds are taken into account individually in the estimation of esds in distances, angles and torsion angles; correlations between esds in cell parameters are only used when they are defined by crystal symmetry. An approximate (isotropic) treatment of cell esds is used for estimating esds involving l.s. planes.

### Fractional atomic coordinates and isotropic or equivalent isotropic displacement parameters (Å^2^)

**Table:**

	*x*	*y*	*z*	*U*_iso_*/*U*_eq_	
C1	0.3350 (2)	−0.0912 (2)	−0.09164 (11)	0.0366 (5)	
C2	0.3233 (3)	0.0078 (3)	−0.13822 (13)	0.0569 (7)	
H2	0.274001	0.087883	−0.127471	0.068*	
C3	0.3836 (3)	−0.0093 (3)	−0.20113 (14)	0.0669 (9)	
H3	0.373766	0.059391	−0.231879	0.080*	
C4	0.4571 (3)	−0.1256 (3)	−0.21835 (13)	0.0604 (8)	
H4	0.496492	−0.136979	−0.260675	0.072*	
C5	0.4716 (4)	−0.2241 (3)	−0.17255 (16)	0.0871 (12)	
H5	0.522325	−0.303681	−0.183478	0.104*	
C6	0.4119 (4)	−0.2078 (3)	−0.10953 (15)	0.0783 (11)	
H6	0.423794	−0.276754	−0.078790	0.094*	
C7	0.3442 (2)	−0.2146 (2)	0.03656 (11)	0.0390 (5)	
C8	0.3086 (3)	−0.3249 (2)	0.04008 (14)	0.0511 (6)	
H8	0.226479	−0.325282	0.022724	0.061*	
C9	0.3955 (3)	−0.4347 (3)	0.06947 (16)	0.0652 (8)	
H9	0.371010	−0.508227	0.072061	0.078*	
C10	0.5178 (4)	−0.4348 (3)	0.09479 (16)	0.0738 (10)	
H10	0.575632	−0.508373	0.114609	0.089*	
C11	0.5544 (4)	−0.3268 (3)	0.09079 (17)	0.0769 (10)	
H11	0.637579	−0.327498	0.107470	0.092*	
C12	0.4686 (3)	−0.2169 (3)	0.06214 (14)	0.0590 (7)	
H12	0.494085	−0.143885	0.059898	0.071*	
C13	0.0449 (2)	0.2538 (2)	0.09219 (12)	0.0405 (5)	
C14	0.1073 (3)	0.3397 (2)	0.06425 (13)	0.0533 (7)	
H14	0.184744	0.313685	0.037036	0.064*	
C15	0.0550 (4)	0.4655 (3)	0.07660 (15)	0.0617 (8)	
H15	0.097124	0.522788	0.056868	0.074*	
C16	−0.0561 (3)	0.5053 (3)	0.11686 (17)	0.0665 (9)	
H16	−0.090233	0.589351	0.124820	0.080*	
C17	−0.1177 (3)	0.4208 (3)	0.1457 (2)	0.0769 (10)	
H17	−0.193003	0.447450	0.174131	0.092*	
C18	−0.0685 (3)	0.2951 (3)	0.13295 (18)	0.0666 (9)	
H18	−0.112536	0.238741	0.152063	0.080*	
C19	0.1400 (2)	0.0176 (2)	0.16260 (11)	0.0383 (5)	
C20	0.0376 (3)	−0.0158 (2)	0.20019 (13)	0.0499 (6)	
H20	−0.044492	−0.010321	0.180707	0.060*	
C21	0.0576 (3)	−0.0575 (3)	0.26715 (14)	0.0600 (8)	
H21	−0.012310	−0.077546	0.292316	0.072*	
C22	0.1797 (4)	−0.0694 (3)	0.29637 (14)	0.0653 (8)	
H22	0.192690	−0.098071	0.340907	0.078*	
C23	0.2815 (4)	−0.0387 (4)	0.25948 (15)	0.0763 (10)	
H23	0.364497	−0.047061	0.278836	0.092*	
C24	0.2617 (3)	0.0051 (3)	0.19290 (13)	0.0612 (8)	
H24	0.331704	0.026306	0.168395	0.073*	
C25	0.2659 (2)	0.0574 (2)	0.02992 (11)	0.0381 (5)	
H25A	0.344711	0.032317	0.058288	0.046*	
H25B	0.270235	0.126984	−0.001278	0.046*	
C26	0.3245 (2)	0.4602 (2)	0.34333 (10)	0.0354 (5)	
C27	0.3270 (3)	0.5749 (2)	0.31347 (11)	0.0431 (5)	
H27	0.366858	0.624437	0.335022	0.052*	
C28	0.2698 (3)	0.6171 (3)	0.25075 (12)	0.0519 (6)	
H28	0.273664	0.693687	0.230569	0.062*	
C29	0.2082 (3)	0.5459 (3)	0.21908 (13)	0.0556 (7)	
H29	0.170546	0.574364	0.177557	0.067*	
C30	0.2021 (3)	0.4322 (3)	0.24871 (13)	0.0588 (7)	
H30	0.158978	0.384751	0.227560	0.071*	
C31	0.2609 (3)	0.3886 (3)	0.31052 (13)	0.0498 (6)	
H31	0.257827	0.311398	0.330099	0.060*	
C32	0.4930 (2)	0.2365 (2)	0.40974 (11)	0.0358 (5)	
C33	0.4360 (3)	0.1384 (2)	0.42135 (13)	0.0501 (6)	
H33	0.346200	0.154106	0.437152	0.060*	
C34	0.5131 (4)	0.0167 (3)	0.40936 (14)	0.0647 (9)	
H34	0.474194	−0.048249	0.416968	0.078*	
C35	0.6441 (4)	−0.0074 (3)	0.38673 (16)	0.0728 (10)	
H35	0.694871	−0.088757	0.378823	0.087*	
C36	0.7023 (3)	0.0881 (3)	0.37543 (17)	0.0733 (9)	
H36	0.792447	0.071283	0.360108	0.088*	
C37	0.6264 (3)	0.2098 (3)	0.38689 (14)	0.0544 (7)	
H37	0.666269	0.274091	0.379018	0.065*	
C38	0.1356 (2)	0.6455 (2)	0.53378 (11)	0.0360 (5)	
C39	0.1005 (3)	0.7093 (2)	0.47162 (13)	0.0479 (6)	
H39	0.146687	0.675909	0.433590	0.057*	
C40	−0.0029 (3)	0.8220 (3)	0.46614 (16)	0.0612 (8)	
H40	−0.026056	0.863316	0.424432	0.073*	
C41	−0.0713 (3)	0.8731 (3)	0.52173 (18)	0.0639 (8)	
H41	−0.140749	0.948578	0.517661	0.077*	
C42	−0.0370 (3)	0.8124 (3)	0.58373 (16)	0.0606 (8)	
H42	−0.082373	0.847815	0.621487	0.073*	
C43	0.0650 (3)	0.6987 (2)	0.58991 (14)	0.0480 (6)	
H43	0.086454	0.657635	0.631801	0.058*	
C44	0.2435 (2)	0.4289 (2)	0.62126 (11)	0.0387 (5)	
C45	0.1282 (3)	0.3879 (3)	0.62890 (14)	0.0562 (7)	
H45	0.070376	0.399796	0.593281	0.067*	
C46	0.0982 (4)	0.3308 (3)	0.68751 (18)	0.0786 (11)	
H46	0.019450	0.305798	0.691747	0.094*	
C47	0.1828 (5)	0.3100 (3)	0.73989 (19)	0.0947 (15)	
H47	0.162193	0.270075	0.779579	0.114*	
C48	0.2989 (5)	0.3480 (4)	0.73431 (16)	0.0967 (14)	
H48	0.357669	0.332119	0.769833	0.116*	
C49	0.3284 (3)	0.4109 (3)	0.67465 (13)	0.0650 (8)	
H49	0.404421	0.440022	0.671194	0.078*	
C50	0.2543 (2)	0.3965 (2)	0.48070 (11)	0.0368 (5)	
H50A	0.261637	0.313377	0.501122	0.044*	
H50B	0.167960	0.430495	0.458281	0.044*	
P1	0.24044 (6)	−0.07535 (5)	−0.01129 (3)	0.03232 (12)	
P2	0.10162 (6)	0.08777 (5)	0.07737 (3)	0.03266 (12)	
P3	0.40258 (6)	0.39950 (5)	0.42502 (3)	0.02984 (12)	
P4	0.28068 (6)	0.50279 (5)	0.54122 (3)	0.03042 (12)	
Cl1	0.01479 (6)	0.17901 (5)	−0.07470 (3)	0.04094 (13)	
Cl2	0.42550 (6)	0.71029 (5)	0.44483 (3)	0.03774 (12)	
Ru1	0.000000	0.000000	0.000000	0.02905 (6)	
Ru2	0.500000	0.500000	0.500000	0.02626 (6)	

### Atomic displacement parameters (Å^2^)

**Table:**

	*U*^11^	*U*^22^	*U*^33^	*U*^12^	*U*^13^	*U*^23^
C1	0.0352 (12)	0.0391 (12)	0.0350 (11)	−0.0106 (10)	−0.0004 (9)	−0.0016 (9)
C2	0.0528 (16)	0.0485 (15)	0.0529 (15)	0.0044 (12)	0.0132 (13)	0.0080 (12)
C3	0.0637 (19)	0.070 (2)	0.0488 (15)	−0.0018 (16)	0.0118 (14)	0.0176 (14)
C4	0.0621 (18)	0.075 (2)	0.0422 (14)	−0.0184 (16)	0.0144 (13)	−0.0087 (14)
C5	0.131 (3)	0.0533 (19)	0.0632 (19)	−0.012 (2)	0.045 (2)	−0.0116 (15)
C6	0.116 (3)	0.0436 (16)	0.0585 (17)	−0.0058 (17)	0.0352 (19)	0.0013 (13)
C7	0.0372 (12)	0.0372 (12)	0.0347 (11)	−0.0011 (10)	0.0028 (9)	0.0016 (9)
C8	0.0405 (14)	0.0420 (14)	0.0638 (16)	−0.0052 (11)	0.0059 (12)	0.0038 (12)
C9	0.064 (2)	0.0419 (15)	0.077 (2)	−0.0039 (14)	0.0117 (16)	0.0098 (14)
C10	0.074 (2)	0.059 (2)	0.0662 (19)	0.0114 (17)	−0.0164 (17)	0.0121 (15)
C11	0.068 (2)	0.069 (2)	0.080 (2)	0.0028 (17)	−0.0368 (18)	0.0011 (18)
C12	0.0553 (17)	0.0492 (16)	0.0672 (18)	−0.0062 (13)	−0.0228 (14)	0.0015 (13)
C13	0.0448 (14)	0.0334 (12)	0.0441 (12)	−0.0103 (10)	−0.0084 (11)	−0.0082 (10)
C14	0.0717 (19)	0.0393 (14)	0.0538 (15)	−0.0231 (13)	0.0008 (14)	−0.0077 (12)
C15	0.090 (2)	0.0400 (15)	0.0640 (18)	−0.0303 (15)	−0.0173 (17)	−0.0013 (13)
C16	0.072 (2)	0.0369 (15)	0.087 (2)	−0.0048 (14)	−0.0267 (18)	−0.0146 (15)
C17	0.060 (2)	0.0483 (17)	0.116 (3)	−0.0013 (15)	0.0073 (19)	−0.0279 (18)
C18	0.0529 (17)	0.0387 (15)	0.106 (3)	−0.0092 (13)	0.0121 (17)	−0.0152 (15)
C19	0.0438 (13)	0.0348 (12)	0.0360 (11)	−0.0103 (10)	−0.0002 (10)	−0.0055 (9)
C20	0.0508 (15)	0.0503 (15)	0.0486 (14)	−0.0159 (12)	0.0009 (12)	−0.0018 (12)
C21	0.075 (2)	0.0557 (17)	0.0475 (15)	−0.0205 (15)	0.0135 (15)	−0.0010 (13)
C22	0.091 (2)	0.0616 (19)	0.0383 (14)	−0.0152 (17)	−0.0021 (15)	−0.0011 (13)
C23	0.073 (2)	0.107 (3)	0.0519 (17)	−0.031 (2)	−0.0180 (16)	0.0018 (18)
C24	0.0567 (18)	0.088 (2)	0.0451 (14)	−0.0320 (16)	−0.0075 (13)	0.0009 (14)
C25	0.0364 (12)	0.0382 (12)	0.0413 (12)	−0.0137 (10)	0.0010 (10)	−0.0041 (10)
C26	0.0303 (11)	0.0437 (13)	0.0319 (10)	−0.0081 (9)	−0.0046 (9)	−0.0082 (9)
C27	0.0464 (14)	0.0449 (14)	0.0394 (12)	−0.0139 (11)	−0.0052 (11)	−0.0066 (10)
C28	0.0587 (17)	0.0541 (16)	0.0403 (13)	−0.0136 (13)	−0.0071 (12)	0.0019 (11)
C29	0.0530 (16)	0.0711 (19)	0.0381 (13)	−0.0099 (14)	−0.0122 (12)	−0.0043 (13)
C30	0.0605 (18)	0.073 (2)	0.0493 (15)	−0.0239 (15)	−0.0194 (13)	−0.0145 (14)
C31	0.0535 (16)	0.0523 (15)	0.0484 (14)	−0.0203 (13)	−0.0157 (12)	−0.0057 (12)
C32	0.0388 (12)	0.0335 (11)	0.0347 (11)	−0.0077 (9)	−0.0073 (9)	−0.0084 (9)
C33	0.0634 (17)	0.0399 (13)	0.0488 (14)	−0.0172 (12)	−0.0001 (13)	−0.0069 (11)
C34	0.102 (3)	0.0374 (14)	0.0540 (16)	−0.0188 (16)	−0.0147 (17)	−0.0020 (12)
C35	0.086 (2)	0.0451 (17)	0.070 (2)	0.0146 (16)	−0.0247 (18)	−0.0207 (15)
C36	0.0490 (17)	0.069 (2)	0.092 (2)	0.0055 (15)	−0.0052 (16)	−0.0332 (18)
C37	0.0438 (15)	0.0531 (16)	0.0671 (17)	−0.0100 (12)	−0.0006 (13)	−0.0255 (13)
C38	0.0271 (11)	0.0360 (12)	0.0471 (12)	−0.0115 (9)	−0.0015 (9)	−0.0075 (10)
C39	0.0362 (13)	0.0530 (15)	0.0521 (14)	−0.0105 (11)	−0.0027 (11)	−0.0006 (12)
C40	0.0413 (15)	0.0557 (17)	0.079 (2)	−0.0077 (13)	−0.0069 (14)	0.0149 (15)
C41	0.0394 (15)	0.0412 (15)	0.106 (3)	−0.0059 (12)	−0.0016 (16)	−0.0015 (16)
C42	0.0470 (16)	0.0516 (16)	0.082 (2)	−0.0097 (13)	0.0118 (15)	−0.0253 (16)
C43	0.0430 (14)	0.0439 (14)	0.0562 (15)	−0.0097 (11)	0.0026 (12)	−0.0129 (12)
C44	0.0390 (12)	0.0374 (12)	0.0365 (11)	−0.0075 (10)	0.0071 (10)	−0.0042 (9)
C45	0.0570 (17)	0.0560 (16)	0.0604 (16)	−0.0260 (14)	0.0141 (14)	−0.0054 (13)
C46	0.097 (3)	0.0551 (19)	0.082 (2)	−0.0276 (18)	0.049 (2)	−0.0074 (17)
C47	0.131 (4)	0.065 (2)	0.065 (2)	−0.006 (2)	0.053 (2)	0.0077 (17)
C48	0.112 (3)	0.112 (3)	0.0382 (16)	0.006 (3)	−0.0014 (19)	0.0040 (18)
C49	0.0579 (18)	0.091 (2)	0.0390 (13)	−0.0112 (16)	0.0033 (13)	−0.0053 (14)
C50	0.0337 (12)	0.0424 (12)	0.0398 (11)	−0.0178 (10)	0.0023 (9)	−0.0113 (10)
P1	0.0305 (3)	0.0294 (3)	0.0349 (3)	−0.0063 (2)	0.0006 (2)	−0.0012 (2)
P2	0.0336 (3)	0.0294 (3)	0.0359 (3)	−0.0102 (2)	−0.0008 (2)	−0.0045 (2)
P3	0.0291 (3)	0.0318 (3)	0.0302 (3)	−0.0100 (2)	−0.0023 (2)	−0.0067 (2)
P4	0.0276 (3)	0.0332 (3)	0.0318 (3)	−0.0104 (2)	0.0005 (2)	−0.0055 (2)
Cl1	0.0424 (3)	0.0307 (3)	0.0474 (3)	−0.0097 (2)	0.0013 (2)	0.0033 (2)
Cl2	0.0449 (3)	0.0307 (3)	0.0378 (3)	−0.0114 (2)	−0.0061 (2)	−0.0006 (2)
Ru1	0.02943 (13)	0.02389 (12)	0.03301 (12)	−0.00670 (9)	0.00029 (9)	−0.00270 (9)
Ru2	0.02574 (12)	0.02694 (12)	0.02692 (11)	−0.00852 (9)	−0.00124 (9)	−0.00377 (9)

### Geometric parameters (Å, º)

**Table:**

C1—C2	1.371 (3)	C27—C28	1.405 (3)
C1—C6	1.381 (4)	C27—H27	0.9300
C1—P1	1.844 (2)	C28—C29	1.373 (4)
C2—C3	1.389 (4)	C28—H28	0.9300
C2—H2	0.9300	C29—C30	1.379 (4)
C3—C4	1.365 (4)	C29—H29	0.9300
C3—H3	0.9300	C30—C31	1.396 (3)
C4—C5	1.355 (4)	C30—H30	0.9300
C4—H4	0.9300	C31—H31	0.9300
C5—C6	1.388 (4)	C32—C37	1.376 (4)
C5—H5	0.9300	C32—C33	1.393 (3)
C6—H6	0.9300	C32—P3	1.835 (2)
C7—C8	1.389 (3)	C33—C34	1.395 (4)
C7—C12	1.393 (4)	C33—H33	0.9300
C7—P1	1.829 (2)	C34—C35	1.354 (5)
C8—C9	1.389 (4)	C34—H34	0.9300
C8—H8	0.9300	C35—C36	1.374 (5)
C9—C10	1.378 (5)	C35—H35	0.9300
C9—H9	0.9300	C36—C37	1.390 (4)
C10—C11	1.370 (5)	C36—H36	0.9300
C10—H10	0.9300	C37—H37	0.9300
C11—C12	1.380 (4)	C38—C39	1.396 (3)
C11—H11	0.9300	C38—C43	1.396 (3)
C12—H12	0.9300	C38—P4	1.832 (2)
C13—C18	1.377 (4)	C39—C40	1.387 (4)
C13—C14	1.379 (3)	C39—H39	0.9300
C13—P2	1.829 (2)	C40—C41	1.371 (4)
C14—C15	1.394 (4)	C40—H40	0.9300
C14—H14	0.9300	C41—C42	1.380 (4)
C15—C16	1.353 (4)	C41—H41	0.9300
C15—H15	0.9300	C42—C43	1.387 (4)
C16—C17	1.365 (5)	C42—H42	0.9300
C16—H16	0.9300	C43—H43	0.9300
C17—C18	1.395 (4)	C44—C49	1.382 (4)
C17—H17	0.9300	C44—C45	1.389 (4)
C18—H18	0.9300	C44—P4	1.827 (2)
C19—C24	1.382 (4)	C45—C46	1.362 (4)
C19—C20	1.389 (3)	C45—H45	0.9300
C19—P2	1.838 (2)	C46—C47	1.361 (6)
C20—C21	1.395 (4)	C46—H46	0.9300
C20—H20	0.9300	C47—C48	1.377 (6)
C21—C22	1.376 (4)	C47—H47	0.9300
C21—H21	0.9300	C48—C49	1.408 (4)
C22—C23	1.363 (4)	C48—H48	0.9300
C22—H22	0.9300	C49—H49	0.9300
C23—C24	1.393 (4)	C50—P3	1.854 (2)
C23—H23	0.9300	C50—P4	1.866 (2)
C24—H24	0.9300	C50—H50A	0.9700
C25—P2	1.852 (2)	C50—H50B	0.9700
C25—P1	1.862 (2)	P1—Ru1	2.3623 (12)
C25—H25A	0.9700	P2—Ru1	2.3573 (9)
C25—H25B	0.9700	P3—Ru2	2.3882 (9)
C26—C27	1.381 (3)	P4—Ru2	2.3529 (11)
C26—C31	1.402 (3)	Cl1—Ru1	2.4426 (10)
C26—P3	1.852 (2)	Cl2—Ru2	2.4375 (11)
			
C2—C1—C6	117.1 (2)	C34—C35—C36	120.1 (3)
C2—C1—P1	122.55 (18)	C34—C35—H35	119.9
C6—C1—P1	120.11 (19)	C36—C35—H35	119.9
C1—C2—C3	121.3 (3)	C35—C36—C37	120.0 (3)
C1—C2—H2	119.3	C35—C36—H36	120.0
C3—C2—H2	119.3	C37—C36—H36	120.0
C4—C3—C2	120.8 (3)	C32—C37—C36	120.8 (3)
C4—C3—H3	119.6	C32—C37—H37	119.6
C2—C3—H3	119.6	C36—C37—H37	119.6
C5—C4—C3	118.7 (3)	C39—C38—C43	118.3 (2)
C5—C4—H4	120.7	C39—C38—P4	120.19 (18)
C3—C4—H4	120.7	C43—C38—P4	121.31 (19)
C4—C5—C6	120.9 (3)	C40—C39—C38	120.5 (3)
C4—C5—H5	119.5	C40—C39—H39	119.8
C6—C5—H5	119.5	C38—C39—H39	119.8
C1—C6—C5	121.2 (3)	C41—C40—C39	120.5 (3)
C1—C6—H6	119.4	C41—C40—H40	119.7
C5—C6—H6	119.4	C39—C40—H40	119.7
C8—C7—C12	118.8 (2)	C40—C41—C42	120.0 (3)
C8—C7—P1	119.9 (2)	C40—C41—H41	120.0
C12—C7—P1	120.7 (2)	C42—C41—H41	120.0
C7—C8—C9	120.2 (3)	C41—C42—C43	120.2 (3)
C7—C8—H8	119.9	C41—C42—H42	119.9
C9—C8—H8	119.9	C43—C42—H42	119.9
C10—C9—C8	120.1 (3)	C42—C43—C38	120.6 (3)
C10—C9—H9	119.9	C42—C43—H43	119.7
C8—C9—H9	119.9	C38—C43—H43	119.7
C11—C10—C9	120.1 (3)	C49—C44—C45	118.7 (2)
C11—C10—H10	120.0	C49—C44—P4	121.6 (2)
C9—C10—H10	120.0	C45—C44—P4	119.7 (2)
C10—C11—C12	120.4 (3)	C46—C45—C44	121.3 (3)
C10—C11—H11	119.8	C46—C45—H45	119.3
C12—C11—H11	119.8	C44—C45—H45	119.3
C11—C12—C7	120.5 (3)	C47—C46—C45	120.4 (4)
C11—C12—H12	119.8	C47—C46—H46	119.8
C7—C12—H12	119.8	C45—C46—H46	119.8
C18—C13—C14	118.5 (2)	C46—C47—C48	120.2 (3)
C18—C13—P2	117.6 (2)	C46—C47—H47	119.9
C14—C13—P2	124.0 (2)	C48—C47—H47	119.9
C13—C14—C15	120.3 (3)	C47—C48—C49	119.8 (4)
C13—C14—H14	119.8	C47—C48—H48	120.1
C15—C14—H14	119.8	C49—C48—H48	120.1
C16—C15—C14	120.9 (3)	C44—C49—C48	119.5 (3)
C16—C15—H15	119.5	C44—C49—H49	120.3
C14—C15—H15	119.5	C48—C49—H49	120.3
C15—C16—C17	119.4 (3)	P3—C50—P4	96.88 (10)
C15—C16—H16	120.3	P3—C50—H50A	112.4
C17—C16—H16	120.3	P4—C50—H50A	112.4
C16—C17—C18	120.6 (3)	P3—C50—H50B	112.4
C16—C17—H17	119.7	P4—C50—H50B	112.4
C18—C17—H17	119.7	H50A—C50—H50B	109.9
C13—C18—C17	120.3 (3)	C7—P1—C1	100.18 (10)
C13—C18—H18	119.8	C7—P1—C25	104.13 (11)
C17—C18—H18	119.8	C1—P1—C25	109.64 (10)
C24—C19—C20	118.2 (2)	C7—P1—Ru1	123.18 (8)
C24—C19—P2	123.36 (19)	C1—P1—Ru1	124.30 (8)
C20—C19—P2	118.27 (19)	C25—P1—Ru1	93.06 (8)
C19—C20—C21	120.1 (3)	C13—P2—C19	100.37 (11)
C19—C20—H20	119.9	C13—P2—C25	107.50 (11)
C21—C20—H20	119.9	C19—P2—C25	107.39 (11)
C22—C21—C20	120.8 (3)	C13—P2—Ru1	123.41 (8)
C22—C21—H21	119.6	C19—P2—Ru1	122.87 (8)
C20—C21—H21	119.6	C25—P2—Ru1	93.46 (8)
C23—C22—C21	119.4 (3)	C32—P3—C26	101.27 (10)
C23—C22—H22	120.3	C32—P3—C50	106.71 (11)
C21—C22—H22	120.3	C26—P3—C50	103.65 (11)
C22—C23—C24	120.3 (3)	C32—P3—Ru2	118.24 (8)
C22—C23—H23	119.9	C26—P3—Ru2	129.47 (8)
C24—C23—H23	119.9	C50—P3—Ru2	94.28 (7)
C19—C24—C23	121.2 (3)	C44—P4—C38	101.93 (11)
C19—C24—H24	119.4	C44—P4—C50	102.81 (11)
C23—C24—H24	119.4	C38—P4—C50	106.93 (11)
P2—C25—P1	93.90 (11)	C44—P4—Ru2	125.32 (8)
P2—C25—H25A	112.9	C38—P4—Ru2	121.47 (8)
P1—C25—H25A	112.9	C50—P4—Ru2	95.10 (7)
P2—C25—H25B	112.9	P2^i^—Ru1—P2	180.0
P1—C25—H25B	112.9	P2^i^—Ru1—P1	109.78 (3)
H25A—C25—H25B	110.4	P2—Ru1—P1	70.22 (3)
C27—C26—C31	118.7 (2)	P2^i^—Ru1—P1^i^	70.21 (3)
C27—C26—P3	121.67 (17)	P2—Ru1—P1^i^	109.79 (3)
C31—C26—P3	119.68 (18)	P1—Ru1—P1^i^	180.0
C26—C27—C28	120.4 (2)	P2^i^—Ru1—Cl1	95.43 (4)
C26—C27—H27	119.8	P2—Ru1—Cl1	84.58 (4)
C28—C27—H27	119.8	P1—Ru1—Cl1	86.47 (3)
C29—C28—C27	120.3 (3)	P1^i^—Ru1—Cl1	93.53 (3)
C29—C28—H28	119.8	P2^i^—Ru1—Cl1^i^	84.57 (4)
C27—C28—H28	119.8	P2—Ru1—Cl1^i^	95.42 (4)
C28—C29—C30	120.2 (2)	P1—Ru1—Cl1^i^	93.53 (3)
C28—C29—H29	119.9	P1^i^—Ru1—Cl1^i^	86.47 (3)
C30—C29—H29	119.9	Cl1—Ru1—Cl1^i^	180.00 (3)
C29—C30—C31	119.8 (3)	P4^ii^—Ru2—P4	180.0
C29—C30—H30	120.1	P4^ii^—Ru2—P3^ii^	71.90 (3)
C31—C30—H30	120.1	P4—Ru2—P3^ii^	108.09 (3)
C30—C31—C26	120.6 (3)	P4^ii^—Ru2—P3	108.09 (3)
C30—C31—H31	119.7	P4—Ru2—P3	71.91 (3)
C26—C31—H31	119.7	P3^ii^—Ru2—P3	180.0
C37—C32—C33	118.4 (2)	P4^ii^—Ru2—Cl2	85.27 (3)
C37—C32—P3	117.49 (19)	P4—Ru2—Cl2	94.73 (3)
C33—C32—P3	124.08 (19)	P3^ii^—Ru2—Cl2	82.43 (3)
C32—C33—C34	120.2 (3)	P3—Ru2—Cl2	97.57 (3)
C32—C33—H33	119.9	P4^ii^—Ru2—Cl2^ii^	94.73 (3)
C34—C33—H33	119.9	P4—Ru2—Cl2^ii^	85.27 (3)
C35—C34—C33	120.4 (3)	P3^ii^—Ru2—Cl2^ii^	97.57 (4)
C35—C34—H34	119.8	P3—Ru2—Cl2^ii^	82.43 (4)
C33—C34—H34	119.8	Cl2—Ru2—Cl2^ii^	180.00 (4)

Symmetry codes: (i) −*x*, −*y*, −*z*; (ii) −*x*+1, −*y*+1, −*z*+1.

### Hydrogen-bond geometry (Å, º)

**Table:**

*D*—H···*A*	*D*—H	H···*A*	*D*···*A*	*D*—H···*A*
C8—H8···Cl1^i^	0.93	2.75	3.302 (3)	119
C34—H34···Cl2^iii^	0.93	2.91	3.813 (3)	165
C2—H2···Cl1	0.93	2.75	3.429 (3)	131
C27—H27···Cl2	0.93	2.66	3.506 (3)	152

Symmetry codes: (i) −*x*, −*y*, −*z*; (iii) *x*, *y*−1, *z*.
